# The Effectiveness of Information Technology-Supported Shared Care for Patients With Chronic Disease: A Systematic Review

**DOI:** 10.2196/jmir.7405

**Published:** 2017-06-22

**Authors:** Laura Kooij, Wim G Groen, Wim H van Harten

**Affiliations:** ^1^ The Netherlands Cancer Institute Division of Psychosocial Research and Epidemiology Amsterdam Netherlands; ^2^ University of Twente Department of Health Technology and Services Research Enschede Netherlands; ^3^ Rijnstate hospital Arnhem Netherlands

**Keywords:** review, integrated healthcare systems, health information systems, chronic disease

## Abstract

**Background:**

In patients with chronic disease, many health care professionals are involved during treatment and follow-up. This leads to fragmentation that in turn may lead to suboptimal care. Shared care is a means to improve the integration of care delivered by various providers, specifically primary care physicians (PCPs) and specialty care professionals, for patients with chronic disease. The use of information technology (IT) in this field seems promising.

**Objective:**

Our aim was to systematically review the literature regarding the effectiveness of IT-supported shared care interventions in chronic disease in terms of provider or professional, process, health or clinical and financial outcomes. Additionally, our aim was to provide an inventory of the IT applications' characteristics that support such interventions.

**Methods:**

PubMed, Scopus, and EMBASE were searched from 2006 to 2015 to identify relevant studies using search terms related to shared care, chronic disease, and IT. Eligible studies were in the English language, and the randomized controlled trials (RCTs), controlled trials, or single group pre-post studies used reported on the effects of IT-supported shared care in patients with chronic disease and cancer. The interventions had to involve providers from both primary and specialty health care. Intervention and IT characteristics and effectiveness—in terms of provider or professional (proximal), process (intermediate), health or clinical and financial (distal) outcomes—were extracted. Risk of bias of (cluster) RCTs was assessed using the Cochrane tool.

**Results:**

The initial search yielded 4167 results. Thirteen publications were used, including 11 (cluster) RCTs, a controlled trial, and a pre-post feasibility study. Four main categories of IT applications were identified: (1) electronic decision support tools, (2) electronic platform with a call-center, (3) electronic health records, and (4) electronic communication applications. Positive effects were found for decision support-based interventions on financial and health outcomes, such as physical activity. Electronic health record use improved PCP visits and reduced rehospitalization. Electronic platform use resulted in fewer readmissions and better clinical outcomes—for example, in terms of body mass index (BMI) and dyspnea. The use of electronic communication applications using text-based information transfer between professionals had a positive effect on the number of PCPs contacting hospitals, PCPs’ satisfaction, and confidence.

**Conclusions:**

IT-supported shared care can improve proximal outcomes, such as confidence and satisfaction of PCPs, especially in using electronic communication applications. Positive effects on intermediate and distal outcomes were also reported but were mixed. Surprisingly, few studies were found that substantiated these anticipated benefits. Studies showed a large heterogeneity in the included populations, outcome measures, and IT applications used. Therefore, a firm conclusion cannot be drawn. As IT applications are developed and implemented rapidly, evidence is needed to test the specific added value of IT in shared care interventions. This is expected to require innovative research methods.

## Introduction

In Europe, 77 % of the disease burden is attributable to chronic diseases. For example, 60 million people live with diabetes [1] and 4-10% suffer from chronic obstructive pulmonary disease (COPD) [2]. Cancer is the leading cause of death in Europe with at least 3 million new cases each year, and cancer survivors are increasingly considered as having a chronic disease [3]. Many health care professionals and various providers are involved during treatment and follow-up of patients with these chronic diseases [3,4]. This inevitably increases fragmentation and can lead to suboptimal care [3]. Coordination of care between multiple professionals caring for patients with chronic disease is essential to guarantee quality of care [4,5]. However, coordination and integration of different professionals is often lacking [3,4]. Shared care is a means to improve integration and is defined as “the joint participation of GPs and hospital consultants in the planned delivery of care for patients with a chronic condition, informed by an enhanced information exchange over and above routine discharge and referral letters” [6]. Shared care can improve care delivery, since it involves a collaboration between primary and specialty care professionals, and this delivery of care is expected to be better than the separation of specialty and primary care [7]. Optimal information exchange between health care professionals is very important for the coordination and continuity of care [8,9]. However, oftentimes information exchange between professionals caring for the same patient is suboptimal [9,10], since professionals lack information [9] or the information is not exchanged on time [10].

The use of information technology (IT) seems promising [10] and is increasingly used to support information exchange [6]. IT can improve information accessibility [4,11-13] and can have a positive effect on safety [14,15]. Additionally, IT can support health care processes and has the potential to improve quality [16] and efficiency of care processes [15,16]. For example, electronic referral can improve the quality of care, access to a professional, and decrease costs [17], and electronic reminders can improve efficiency [4].

An overview of the characteristics and effectiveness of IT-supported shared care interventions is lacking. Previous systematic reviews, such as by Smith et al, provided a total overview of shared care interventions for chronic disease including IT support. They found shared care to be a promising approach but only three IT-supported shared care interventions were reported on. Therefore, there is a need for more evidence, especially as the selected studies were of low methodological quality [7,18]. We presume that since previous reviews [7,18], considerably more IT-supported shared care interventions have been developed and reported on in the literature. Also, IT applications in health care are being developed and implemented at a rapid pace and involve considerable costs. Therefore, we aim to systematically review the state-of-the-art regarding the effectiveness of IT-supported shared care interventions on the care of patients with chronic diseases: diabetes, chronic obstructive pulmonary disease (COPD), (congestive) heart failure, cardiovascular disease (CVD), hypertension, asthma, or cancer. More specifically, we aim to provide an inventory of the effects of shared care, supported by IT, on the care of patients with chronic diseases and to describe the characteristics of the IT applications that support such interventions.

## Methods

### Information Sources and Search Strategy

Studies were identified by searching the literature in EMBASE, Scopus, and PubMed from January 2006 to September 2015. The search consisted of three concepts: (1) shared care, (2) chronic disease, and (3) IT. Several mesh terms were used for these concepts. The full search string is provided in Multimedia Appendix 1. We also checked the reference lists of included articles to detect other relevant studies focusing on (other) chronic diseases (“snowballing method”). As we wanted to provide a total overview of IT-supported shared care interventions, we selected relevant studies from before 2006 from 2 excellent previous reviews (that searched up until 2006) [7,18].

### Eligibility Criteria

For the selection, we used the following eligibility criteria: (1) English-language studies describing a randomized controlled trial (RCT), nonrandomized controlled study or a single-group before and after study; (2) included a shared care intervention; (3) supported by IT; (4) developed specifically for people with a chronic disease: diabetes, COPD, congestive heart failure, CVD, hypertension, or asthma, or cancer; (5) involved health care providers were both primary care physicians (PCPs) operating outside hospitals or physician practices and specialty health care professionals; and (6) study included outcome measures focusing on at least health or clinical, process, provider or professional and financial outcomes.

### Study Selection

The first and second authors independently assessed titles and abstracts focusing on the concepts of shared care, type of disease, and study type. IT was not a criterion for the abstract rejection because it was assumed that IT might only be described in the full texts. In the case of ambiguity or when there was no consensus about the abstracts, the full publication was reviewed by the 2 authors. Disagreement was resolved by discussion; when an issue remained unresolved, the decision of a third reviewer (WvH) was decisive. This selection process was similar for the further selection of full texts.

### Data Extraction

From the selected studies, we report on study characteristics (year, design, measurement time points, and country), patient population (number and type of disease), intervention characteristics (content), IT characteristics (type of application), outcome measures, and effects. The latter were structured according to provider or professional (proximal), process (intermediate), health or clinical and financial (distal) outcomes. These data items were extracted independently by 2 researchers (LK and WG) and disagreement was resolved by discussion.

### Risk of Bias Assessment

We assessed the risk of bias of the included (cluster) RCTs by using the Cochrane risk of bias tool.

The risk of bias was independently assessed by 2 researchers (LK and WG). Disagreement was solved by discussion until consensus was reached. Each aspect and the overall risk of bias of the Cochrane risk of bias tool was graded as high, low, or unclear according to the criteria in the Cochrane handbook [19].

### Synthesis of Results

For the reporting of this systematic review, we used the PRISMA guidelines [20]. Results were synthesized in a qualitative way as there were large differences in the types of intervention, target populations, and outcome measures. Due to the diversity of intervention characteristics and outcomes measures, we could not conduct a meta-analysis.

## Results

### Study Selection

**Figure 1:**
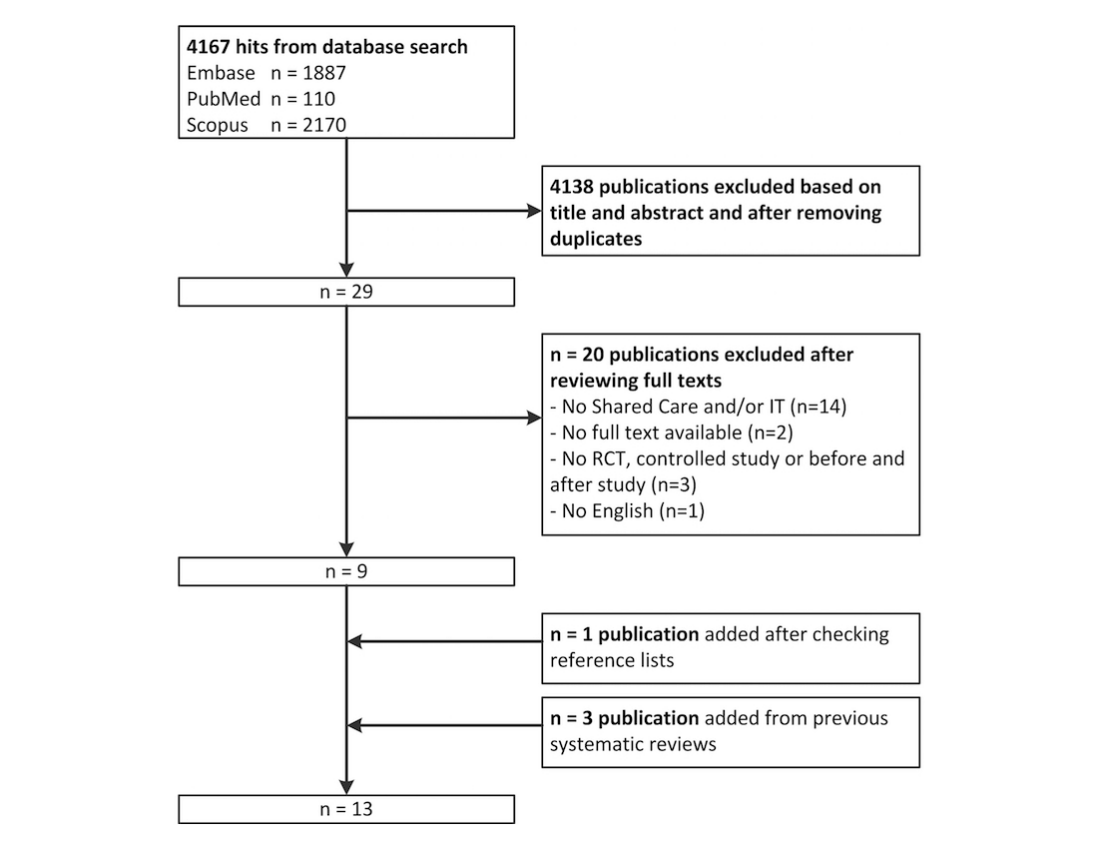
Flowchart of the search and selection procedure.

The primary search yielded 4167 results. After title and abstract selection and the removal of duplicates, 29 papers were read in full text. Nine articles met our inclusion criteria. One additional study was found by reviewing the reference lists, and we identified 3 additional studies from the previous systematic review of Smith et al [7,18]. Reasons for excluding studies were inappropriate study design, no available full text, lack of a shared care intervention, and/or lack of IT support. Figure 1 gives a detailed overview of the study selection procedure.

### Study Characteristics

In total, we included 8 RCTs, 3 cluster RCTs, 1 controlled trial, and 1 pre-post feasibility study. The 13 manuscripts described 11 unique studies. Two papers by Casas et al [21] and Garcia-Aymerich et al [22] described the same intervention but with different patient populations and outcome measures. Lalonde et al [23] and Santschi et al [24] both described the same intervention but assessing different outcome measures.

The included studies were conducted in Canada (n=2) [23,24], Italy (n=2) [25,26], Scotland (n=3) [27-29], United States (n=2) [30,31], Australia (n=1) [32], Denmark (n=1) [33], Spain (n=1) [22], and Spain and Belgium (n=1) [21]. The intervention groups were mostly compared with a group receiving usual care [21-25,27,29,30,32,33], with a specialist outpatient and a nurse practitioner clinic [28] or in one case through general correspondence by email [31].

### Patient Population Characteristics

Patient populations included patients with COPD (n=2) [21,22]; chronic kidney disease (CKD; n=2) [23,24]; diabetes (n=3) [25,27,31]; hypertension (n=1) [28]; asthma (n=1) [29]; and multiple conditions, such as heart failure, diabetes, (risk for) CVD (n=1) [26], and cancer (n=2) [32,33]. One study did not specify the target population but considered hospital discharges in general, which included all conditions [30].

### Intervention Characteristics

The intervention characteristics are presented in Multimedia Appendix 2. There was a large variation in the nature of the interventions, IT applications, and the professionals involved. The primary health care providers who participated in the interventions were PCPs or general practitioners (GPs) (n=11) [21,22,25-33] and pharmacists [23,24]. Specialty care professionals included case managers [21,22,26] and specialists [23,24,28,29,31,33]. However, in 4 interventions the type of specialty care professional was not specified [25,27,30,32].

The objectives varied among the included studies. The majority of the interventions aimed to assess the effectiveness of shared care interventions on the level of distal and/or intermediate outcomes. This included (clinical) patient outcomes [22,24,25,31], sometimes in combination with social and economic settings [27,29]. Other objectives were to study the effects on the number of readmissions, GP contacts with the hospital [21,30], or (diabetes) care outcomes [31]. The impact of a pharmaceutical training and communication network on both distal (pharmaceutical opinions and refusals, clinical outcomes) and proximal outcomes (knowledge and satisfaction of pharmacists) were assessed [24]. Proximal outcomes were also assessed, including tailored information provision to GPs [32] and hospital-based case management [33]. One study aimed to evaluate the feasibility, acceptability, and cost-effectiveness of shared care in comparison with other follow-up approaches [28].

### Information Technology (IT) Characteristics

Four types of IT applications can be distinguished: electronic decision support [26,31], electronic health records (EHRs) [25,27-30], an IT platform combined with a call center [21,22], and electronic communication applications [23,24,32,33]. These will be described in more detail in the next section.

#### Electronic Decision Support

The electronic decision support tools were mainly used for care management, specifically for patients with diabetes [31] and (at risk of) CVD, diabetes, or heart failure [26]. A diabetes electronic management system was used to provide PCPs with decision support aimed at reducing cardiovascular risk in diabetes. PCPs received patient-specific and evidence-based information from endocrinologists via secure-email. Based on this information, PCP and patient discussed how to further continue treatment [31]. Decision support was also used to improve care coordination for patients with diabetes, heart failure, and (at risk of) CVD. Therefore, their care managers were provided with notifications and monitoring instruments [26].

#### Electronic Health Records

In one nonrandomized controlled study, PCPs and hospital professionals exchanged information via a connected EHR in care for diabetes patients [25]. In a RCT, a connected EHR provided GPs with information regarding their elderly patients’ hospital discharge [30]. In 3 cases, the EHRs were “synchronized” and therefore used to store information, which was shared between professionals without technology involved (ie, hardcopies were sent via surface mail). GPs send information to secondary care providers, who add this to their EHR. Consequently GPs periodically receive back the latest updated version [27-29].

#### IT Platform Including a Web-Based Call Center

An IT platform was used by case managers to manage COPD patients’ health records. This platform was connected to a call center that was accessible to PCPs and patients to allow them to contact the case manager. This was part of an intervention aimed at improving health or clinical related outcomes [22] and preventing or reducing of hospitalization [21].

#### Electronic Communication Applications

IT applications were used to provide (one-way) electronic communication using text, for example, fax and electronic messaging. This information was provided by specialty care professionals to inform primary care physicians about their patients.

Fax was used to inform GPs about chemotherapy and patient specifics [32]. To improve community pharmacists’ control over medication-related problems related to CKD, the predialysis clinic provided them with medication and clinical information by fax [23,24]. Case managers, specially trained nurses, aimed to improve the coordination and continuity of care for patients with colorectal cancer. They used electronic messaging to inform GPs about their patients, including contact information [33].

### Outcome Measures and Effects

The most striking proximal (professional or provider) [23,32,33], intermediate (process) [21,23,30,31,33], and distal (health or clinical and financial) [22-26,31] results are described for each IT category, and a comprehensive overview is presented in Multimedia Appendix 3.

#### Electronic Decision Support

A decision support tool described in an RCT was used with the aim to improve metabolic and cardiovascular risk factor control, process of care, and costs for diabetes patients [31]. In a pre-post feasibility study, electronic decision support was used to support care managers in their care of patients with CVD or heart failure [26].

##### Health or Clinical and Financial Outcomes

Electronic decision support for case management in a pre-post feasibility study showed multiple statistically significant outcomes, for example, days of physical activity per week increased from 2.5 to 4.2 days (*P*<.01) and time from 19.9 to 32.9 min each time, self-monitoring increased by 20-27%. Body mass index (BMI), low-density lipoprotein (LDL), systolic blood pressure (BP), and total cholesterol decreased by 10-20%. Additionally, survey results indicate high levels of satisfaction among physicians, care managers, and patients [26]. However, Smith et al [31] found a significant difference between intervention and usual care for smoking cessation (96.0%, 343/358 in the intervention; 93.0%, 257/277 in the control group; *P*=.04) and aspirin use (66.0%, 238/358 in the intervention; 52.0%, 145/277 in the control group; *P*=.001). A significant effect on metabolic outcomes was not detected. Lower costs were reported benefiting the intervention group. The total mean costs of the intervention were US $6252 compared with US $8564 for the control group (*P*=.02); the outpatient costs for the intervention were US $1842 and US $2129 for the control group (*P*=.04). However, these costs were not specifically related to diabetes care [31].

#### Electronic Health Records

EHRs were used to (1) share (real-time) data by connecting primary and secondary EHRs [25,30], and (2) synchronize records by collecting professionals’ input and storing patients data [27-29].

##### Provider or Professional Outcomes

Use of an EHR for hypertension patients was compared with specialists’ outpatient- and nurse practitioner (NP) follow-up. Sixty-one percent (90/147) of the GPs had a preference to continue shared care and 32% (47/147) preferred shared care over the usual, outpatient- or NP care [28].

##### Process Outcomes

EHRs were used to inform GPs about hospital discharges. This had no significant effect on the number of PCP visits after discharge nor on rehospitalization rates (18.77%, 351/1870) compared with the control group (19.88%, 356/1791) [30]. The use of “synchronized” EHRs did not seem to affect the number of consultations [27], admissions [27,29], or GP consultations [29] compared with usual care. However, significant effects were noted for the number of patients receiving a complete (medical) review after 2 years (82.4%, 220/267) in comparison with outpatients (54.1%, 146/270) and with nurse practitioner (74.8%, 202/270) follow-ups [28].

##### Health or Clinical and Financial Outcomes

Clinical information about diabetes patients was shared between GPs and hospital professionals. This had a significant positive effect on various clinical outcomes—for example, glycated hemoglobin (HbA1c), BMI, LDL, and cholesterol [25]. However, the use of “synchronized” health records showed no difference with usual care for most patient-related outcomes, such as metabolic control, psychosocial problems [27], or sleep disturbance [29].

#### IT platform and Web-Based Call Center

COPD patients’ care managers were accessible for PCPs and patients via a call center that was an integral part of an IT platform in which care managers could also manage health records [21,22].

##### Health or Clinical and Financial Outcomes

A significant effect on the number of patients without readmissions was detected: 55% (36/65) of patients in the intervention group compared with 33% (30/90) of patients in the control (*P*=.03) [21].

The intervention was also evaluated on a range of clinical, health-related, quality of life and lifestyle aspects; and on self-management medical treatment and patients’ satisfaction. Only statistically significant improvements in dyspnea and BMI were detected. Patients in the intervention had better knowledge of the name of their disease (81%, 17/21 vs 44%, 18/41 in usual care group; *P*=.005), awareness of identification of COPD exacerbations (81%, 17/21 vs 22%, 9/41 in usual care group; *P*<.001), and of exacerbations in early COPD treatment (90%, 19/21 vs 66%, 27/41 in usual care group *P*=.04) than patients receiving usual care—without support from a case manager [22].

#### Electronic Communication Applications

Information was transferred from secondary to primary care using electronic communication applications, for example, fax [23,24,32,33].

##### Provider or Professional Outcomes

Overall, PCPs were satisfied about the interventions and information [23,32,33]. For example, GPs receiving extra information about their chemotherapy patients were more confident (7% difference with usual care, *P*=.03) and more satisfied than GPs receiving only the usual correspondence (10% difference with usual care, *P*=.002) [32]. Jefford et al [32] found no effect for GP knowledge, whereas Lalonde et al [23] found that the knowledge of pharmacist in the intervention group increased by more than 30%.

##### Process Outcomes

The majority of process-related outcomes improved significantly in the included interventions. For example, training combined with a communication network for pharmacists had positive effects on the number of pharmaceutical recommendations [23,24]. GPs were informed by electronic messaging in a care management intervention for patients with colorectal cancer. In the 9 months follow-up period, the case manager intervention showed a decrease in GPs contacting the hospital (*P*=.008), and fewer patients contacted GPs during out-of-hours service (that is not daytime) (*P*=.02) compared with the control group [33].

##### Health or Clinical and Financial Outcomes

An effect on systolic BP, but not on diastolic or BP control, was reported in one study [24].

### Risk of Bias

An overview of the risk of bias is provided in Multimedia Appendix 4. No study was free from the risk of bias. Inherent to the type of intervention blinding either the participants or professionals was not possible. Of the 11 included (cluster) RCTs, 6 studies had adequate random sequence generation; in most cases, computer-generated systems were used. More than half of the studies had a low risk of bias for allocation assessment, mainly because of the use of numbered sealed envelopes. Other aspects that were rated for risk of bias were (1) selective reporting, (2) blinding of outcome assessment, and (3) incomplete outcome data. These items were often not reported, and therefore, score as an unclear risk of bias according to the Cochrane handbook [19].

## Discussion

### Summary of Evidence

We have systematically reviewed 13 studies focusing on IT-supported shared care for patients with a chronic disease. Overall, there seems to be much merit in IT supported shared care interventions.

The reviewed interventions were supported by four main categories of IT applications: (1) electronic decision support systems, (2) electronic platform and call center, (3) EHR, and (4) electronic communication applications. The main findings of these studies are (1) electronic decision support-based interventions showed a significant positive effect on reducing costs; (2) connected EHRs resulted in more PCP visits, less rehospitalization and better clinical outcomes; and (3) the use of an IT platform resulted in fewer readmissions and positive effects on some health or clinical outcomes. However, it failed to show positive effects on quality of life or doctor visits. Additionally, (4) the use of electronic communication applications showed positive results in terms of PCPs’ satisfaction, confidence [32], and the lower number of GPs contacting the hospital [33]. However, effects on GPs’ knowledge were inconsistent [23,32].

As IT often was only a small part of the intervention, it is hard to determine its real added value in shared care. The reviewed studies varied considerably with regard to the type of intervention, the studied patient population, the IT applications used, and the various outcome measures. As a result of this great variation, and because no study was free from the risk of bias, it is difficult to reliably compare the effects found between the various studies or to make valid generalizations about outcomes that hold true for most chronic patients.

The level of advancedness of included IT applications varied and they have evolved over time. The intervention studies conducted in 1994 [27-29] all used an EHR to manage clinical information and shared this (nonelectronically) between professionals. EHRs have evolved into connected systems that ensure real-time information exchange. Examples are the EHRs used in the studies of Gurwitz et al [30] and Carallo et al [25]. Surprisingly, in 2008 and 2011, fax was still used to transfer information from secondary to primary care, and on the other hand innovative electronic decision support systems were used as well [26,31]). Such “intelligent” systems support professionals in their care of patients, for example, by sending automatic alerts or providing tailored advice. Based on this review we regard this as the most advanced IT application to support shared care.

### Comparison With Previous Work

The findings of our review are comparable with previous reviews on shared or integrated care, in the way that these also reported mixed overall results. For example, Smith et al reviewed the effectiveness of shared care studies for patients with chronic disease [7,18]. The results of the included studies were mixed, and therefore, they pose that it was not possible to draw conclusions about the effectiveness of the interventions. Also the reviewed interventions were complex and consisted of multiple elements that precluded attribution of the effects to the different elements. Additionally, in line with our review, the studies were of low methodological quality [7,18].

Ouwens et al [34] reviewed integrated care interventions and also found heterogeneity in patient populations, outcomes, and interventions. Although integrated care appears to be an effective approach, this heterogeneity may lead to incorrect conclusions [34]. A similar conclusion was drawn in the review of Aubin et al on the effects of interventions to improve continuity of follow-up care for cancer patients. In this review, a shared care model was used in 14 of 63 studies, and even though some effects in separate studies were found, no clear conclusions could be drawn because the results were too mixed [35]. Again, just as in the review of Smith et al [7,18], the interventions were complex, which makes it hard to determine which elements of the intervention were effective and which were not. Overall, it seems difficult to determine the real added value of shared care as a result of mixed results and heterogeneity in the included populations and intervention elements.

The use of IT based interventions in these previous reviews was minimal and also a description of the applications and their effects was lacking [7,18]. We found several IT-supported share care interventions but unfortunately, we were unable to draw firm conclusions about the added value of IT because it is not evaluated as a single component.

### Future Research

Nowadays, many IT applications have been or are being developed to support health care processes [16], but despite this, we only found a surprising small number of publications analyzing their effectiveness in a controlled study. The rapid development of IT applications for shared care purposes is currently not underpinned by rigorous studies showing its added value. Although in evidence-based medicine the RCT is regarded as the gold standard design, there may be drawbacks in using this design for evaluating health care IT applications. RCTs are, by nature, time and cost intensive and may not be able to keep up with fast developing technologies. In other words, when the results of a RCT are finally available, the IT may be outdated. Other research designs could provide more information and save time [36] and may better keep up with the rapid development of IT. Another approach to reflect the rapid development of IT is to measure the feasibility of an IT intervention in a smaller population within a larger RCT [37].

The assessment of the risk of bias of the studies indicates that there is room for improvement in several areas. For example, concealment of intervention allocation and the lack of blinding of participants were not clearly described. This can mean that the effects are overestimated, and it may also be due to the type of intervention. In future research, researchers should provide estimates (as blinding is seldom possible) about how likely it is that this will influence the outcomes. The measurements should also be described more accurately and preferably distinguish proximal or intermediate or distal outcomes because the exact mechanism of intervention and effects is often unclear. Also better standardization on outcome assessments by using a framework, such as the chronic care model (CCM) may be useful. This is a framework to improve clinical and functional outcomes for patients suffering from a chronic disease, and IT can support that model. Key elements are clinical information systems, including databases and care protocol systems. But other applications are also increasingly used to share data with patients, such as patient portals and PHRs. These are applications to provide patients with their clinical information and the ability to share this information [38,39]. Patients’ needs are important, and care should be focused on patients’ preferences to improve quality of care [40]. Professionals should work together, by means of a shared care model, to meet the needs of patients [41]. In line with this, the definition of shared care may be open to discussion or other care models may be increasingly relevant.

Future research must adapt to these aspects and developments. It is also relevant to examine the processes and time points for which IT will be most valuable in supporting shared care.

### Limitations

A limitation of this study is the inclusion of “IT” as a search term in the initial search (title or abstract selection). We therefore might have missed studies that were supported by IT but did not mention this in the title or abstract. Furthermore, although we included a broad range of terms in our search, we may not have retrieved all studies that in fact are a shared care intervention. Our search was conducted from 2006 to January 2015, and we added IT-supported shared care studies from before 2006 from the review of Smith et al [7,18] Although unlikely, we might miss relevant studies from before 2006 that were not reviewed by Smith et al [7,18] because they used slightly different search terms.

### Conclusions

Despite the potential benefits of using IT to support shared care in chronic diseases, we found surprisingly few—whether controlled or uncontrolled—studies that substantiated these anticipated benefits. Studies showed a large heterogeneity in the study populations, outcome measures, and IT applications. The reviewed interventions reported many positive effects on (proximal) provider or professionals outcomes (such as GPs’ satisfaction and confidence). To a lesser extent, positive effects on intermediate (GPs contacting the hospital) and distal outcomes (costs and readmissions) were also reported. Nonetheless, a firm conclusion cannot be drawn on the effect of IT-supported shared care — especially its clinical effect. As IT applications for shared care are developed and implemented rapidly, we are in need of more and better evidence on the specific added value of IT in shared care interventions, and this is expected to require innovative research methods.

## Appendix

Multimedia Appendix 1 Search strategy in PubMed.

Multimedia Appendix 2 Study and information technology (IT) characteristics.

Multimedia Appendix 3 Outcome measures and effects; + indicates a positive effect and ‒ indicates a negative effect.

Multimedia Appendix 4 Risk of bias.

## Abbreviations

BMI: body mass index
BP: blood pressure
CKD: chronic kidney disease
COPD: chronic obstructive pulmonary disease
CVD: cardiovascular disease
EHR: electronic health record
GP: general practitioner
IT: information technology
LDL: low-density lipoprotein
NP: nurse practitioner
PCP: primary care physician
PHR: personal health record
RCT: randomized controlled trial
