# Supplementary material for: The Effectiveness of Information Technology-Supported Shared Care for Patients With Chronic Disease: A Systematic Review
Source: J Med Internet Res. 2017 Jun 22;19(6):e221. doi: 10.2196/jmir.7405 (PMC5500776; doi:10.2196/jmir.7405)
Supplement: Multimedia Appendix 1 [file jmir_v19i6e221_app1.pdf]

**#4 “Study type”**

((random*[tiab] AND (controlled[tiab] OR control[tiab] OR placebo[tiab] OR versus[tiab] OR vs[tiab] OR group[tiab] OR groups[tiab] OR comparison[tiab] OR compared[tiab] OR arm[tiab] OR arms[tiab] OR crossover[tiab] OR cross-over[tiab]) AND (trial[tiab] OR study[tiab])) OR ((single[tiab] OR double[tiab] OR triple[tiab]) AND (masked[tiab] OR blind*[tiab]))) OR ((random*[ot] AND (controlled[ot] OR control[ot] OR placebo[ot] OR versus[ot] OR vs[ot] OR group[ot] OR groups[ot] OR comparison[ot] OR compared[ot] OR arm[ot] OR arms[ot] OR crossover[ot] OR cross-over[ot]) AND (trial[ot] OR study[ot])) OR ((single[ot] OR double[ot] OR triple[ot]) AND (masked[ot] OR blind*[ot]))) OR before and after stud* [tiab] OR "Randomized Controlled Trials as Topic"[Mesh] OR "Interrupted Time Series Analysis"[Mesh] OR ITS stud* [tiab] OR interrupted time ser* [tiab] OR "Controlled Clinical Trials as Topic"[Mesh] OR "Controlled Clinical Trial" [Publication Type] OR "Non-Randomized Controlled Trials as Topic"[Mesh]

**#3 “Cancer and other chronic diseases”**

((neoplasms [mesh] OR cancer* [tiab] OR tumor* [tiab] OR tumour* [tiab] OR neoplasm* [tiab] OR malignan* [tiab]) OR (cancer patient* [tiab] OR cancer survivor* [tiab] OR "Pulmonary Disease, Chronic Obstructive"[Mesh] OR COPD [tiab] OR COAD [tiab] OR (chronic obstructive [tiab] AND (airway [tiab] OR lung [tiab] OR pulmonary [tiab])) OR "Diabetes Mellitus"[Mesh] OR "Diabetes Mellitus, Type 1"[Mesh] OR "Diabetes Mellitus, Type 2"[Mesh] OR diabet* [tiab] OR MODY [tiab] OR NIDDM [tiab] OR IDDM [tiab] OR "heart failure" OR "cardiovascular disease") OR "Asthma"[Mesh] OR asthma* [tiab]) OR ("Hypertension"[Mesh] OR ((High [tiab] OR higher [tiab] OR highest [tiab]) AND blood pressur* [tiab]) OR hypertens* [tiab])

**#2 “Shared Care”**

(delivery of health care, integrated [mesh] OR ((shar* [tiab] OR integrat* [tiab] OR cooperat* [tiab] OR integrat* [tiab] OR collaborat* [tiab] OR link* [tiab] OR exchange* [tiab]) AND (care [tiab]))) AND ((general practitioners [mesh] OR general practice physician* [tiab] OR gp [tiab] OR gps [tiab] OR family doctor* [tiab] OR family physician* [tiab] OR primary health care [mesh] OR primary health care [tiab] OR primary care [tiab]) OR (secondary care [mesh] OR secondary care [tiab] OR secondary health care [tiab] OR hospitals [mesh] OR hospital* [tiab]) OR (Tertiary Healthcare [mesh] OR (tertiar* [tiab] AND (healthcar* [tiab] OR care [tiab] OR caring [tiab]))))

**#1 “Information Technology”**

medical informatics [mesh] OR medical informatic* [tiab] OR information systems [mesh] OR medical records [mesh] OR computer technolog* [tiab] OR information management [mesh] OR information and communication technology [mesh] OR information system* [tiab] OR medical

records systems, computerized [mesh] OR information storage and retrieval [mesh] OR electronic health records [mesh] OR electronic health record* [tiab] OR EHR [tiab] OR EMR [tiab] OR ict [tiab] OR it [tiab] OR systems integration [mesh] OR information exchange [tiab] OR medical records [mesh] OR information dissemination [mesh] OR data integration [tiab] OR information management [mesh]

*Comparable search strategies were performed in Embase and Scopus. Specific features and requirements of each database were taken into account.*
