# Supplementary material for: The Effectiveness of Information Technology-Supported Shared Care for Patients With Chronic Disease: A Systematic Review
Source: J Med Internet Res. 2017 Jun 22;19(6):e221. doi: 10.2196/jmir.7405 (PMC5500776; doi:10.2196/jmir.7405)
Supplement: Multimedia Appendix 2 [file jmir_v19i6e221_app2.pdf]

Study	Design				Intervention characteristics and supporting information technology (in italics)
	Patient target population	Measure-ment time points	Control group	Outcome measures	
Ciccone (2010) [26], pre-post-feasibility study	CVD[a], diabetes, heart failure and/or risk of CVD patients (n=1160)	Baseline, 6, 12, and 18 months	Not applicable	Feasibility and effectiveness in terms of quality of life, therapy adherence, clinical outcomes (BP[b], cholesterol, and glycosylated hemoglobin blood level)	*"Evaluate effectiveness of a disease and care management program and case managers".*<br>- Patient is part of health care team including specialists, GPs[c] and care managers<br>- Care Managers are appointed to GPs<br>- Personal patient care plan<br>- Care managers used an *evidence-based decision support tool* including, for example, health record, notifications related to patients' health

					situation, monitoring, and patient information materials.
Smith (2008) [31], cluster RCT[d]	Physicians (n=97)[e] and diabetes patients (n=639)	Baseline and follow-up: 21 months (mean; 3-36)	Control group: standard information about cardiovascu-lar risk reduction via email	Process of diabetes care, metabolic and cardiovascular risk factor control, and costs	*"To assess the effects of specialist telemedicine intervention on diabetes care outcomes"*<br>- Endocrinolo-gist received medical data from *DEMs*[f] and EHR[g]. Based on this information they could write a tailored advice regarding cardiovascular risk using a *Web-form.* Additionally evidence based information was selected from the *digital library.*<br>- Advice and evidence based messages were sent via *secure-email* to primary care

					(automatic) 48 hours before patients' visit. They could also pick the message up via the DEMS.<br>- Primary care and patient decided how to continue after receiving the information.
Carallo (2015) [25], controlled study (1:2)	Diabetes mellitus type 2 patients (n=312)	Baseline and 1 year	Usual care: follow-up by hospital professionals (quarterly). GPs are informed by letter	Efficacy of the integrated care model in respect of clinical care	*"To verify the efficacy of an integrated care model including GPs empowerment and use of a Web-based EHR in relation to usual care in a clinical setting"*.<br>- Clinical care management shared between GPs and hospital professionals<br>- *Connected EHR* to exchange clinical information<br>- Diabetes type 2 training for GPs<br>- Follow up by

					both GP (quarterly) and hospital professionals (annually).
Gurwitz (2014) [30], RCT	Elderly patients (>65) (all conditions included); hospital discharges (n=3661)	At least six months after end of study	Usual care: follow up at discharge	Primary care visits in 7-, 14-, and 30-day periods after hospital discharge and rehospitaliza-tion within 30 days	*"To assess the effect of EHR-based transitional care intervention on having an outpatient visit with a primary care provider after discharge on being rehospitalized within 30 days of discharge"*<br>- Use of *EHR* to inform GPs about their patients' hospital discharge<br>- GPs received extra medication related information and notification for planning a post hospitalization visit<br>- PCP[h] staff received a message to

					plan a visit with the PCP (except when EHR shows that visit is already planned).
DICE [27], RCT	Diabetes patients—insulin and non-insulin treated (n=274)	Baseline and 2 years	Usual care: patients were seen approxima-tely every 4 months and received (computer generated) reminder letters about regular appointments	Metabolic control, psychosocial status, knowledge, wellbeing and treatment satisfaction, beliefs and control, disruption of normal activities, numbers of consultations and admissions, frequency of metabolic monitoring, and costs	*"Evaluated effectiveness and efficiency of computer coordinated integrated care for insulin and non-insulin treated patients"*<br>- 3 or 4 monthly GP and annually hospital visits.<br>- Integrated care guidelines for GPs<br>- *Computer-based patient record:* to notify GP (patient consults and clinical information) and patients (to make GP appointment) and for coordination of patient records. GP added

					relevant information after a consult to the record, sent it back to hospital where the hospital updated computerized record and returned it to GP.
Drummond [29], RCT (2x2x2; integrated or conven-tional outpatient care; peak flow self-monitoring or usual monitoring; enhanced or usual education)	Patients with asthma (n=712) visiting chest outpatient clinics	Baseline and 1 year	Usual care: 3 monthly visits at outpatient clinical. Receive clinical questionnaire before visit to give to specialist	Number of prescriptions for bronchodilators and inhaled steroids, use of oral steroids, general practice consultations, hospital admissions, sleep disturbance and other restrictions on normal activity; psychological aspects; patient satisfaction and costs	*"To evaluated in clinical, social, and economic terms, the effectiveness of integrated care"*<br>- Annually review of patients records by chest physicians using *computer-based patient record*<br>- 3 monthly visits to GP<br>- Computer generated questionnaire sent to patients and GP<br>GP sends all clinical documents to hospital

					professional who adds information to patient computerized record. GP receives a copy including advice for changes in care.
McGhee (1994) [28], RCT (3 groups)[h]	Patients with (controlled) hypertension (n=831)	Baseline and 2 years	Outpatient care and nurse practitioner clinic care[h]	Effectiveness (number of patients with complete review after 2 years), acceptability (eg, preferences and (dis) advantages), and costs	*"To investigate the feasibility, acceptability and cost effectiveness of shared general practitioner – hospital care for well-controlled hypertensive patients in an urban area by comparing this group with a specialist outpatient clinic and nurse practitioner clinic."*<br>- Shared care between GP, specialist, patient and laboratory with determined roles.<br>- Annually patient review by GP

					- Computerized database used to create medical record (two pages) for GP and patient record summary ("personal health booklet")<br>- After consult: GP sent medical record, results of clinical exams and patient-held record to shared care registry<br>- Results reviews by staff using a protocol and marked abnormalities are reviewed by a specialist<br>- Updated medical record including letter is sent back to GP.
Casas (2006) [21], RCT (1:1.5)	COPD[i] patients (n=155)	1, 3, 6, 9, and 12 months	Usual care without additional support	Primary: hospital readmission. Secondary: mortality and	*Assess the effect of an integrated care intervention, supported by*

				utilization of health care resources	*ICT[j], on prevention of hospitalizations*<br>- Patient assessment at discharge,<br>- Self-management program for patients<br>- Patient tailored care plan shared between case manager and primary care professionals<br>- *IT[k] platform* for case management to manage health records including *Web-based call center* to contact case manager.<br>Follow up: specialized nurse and primary care team (Barcelona) and GP (Leuven).
Garcia-Aymerich (2007) [22], RCT (1:2 ratio)	COPD patients (n=113)	Baseline, 6, and 12 months	Control group: patients received usual care without	Effectiveness: clinical, health-related quality of life, lifestyle, self-management,	*"To assess the effectiveness of an integrate care intervention to enhance clinical*

			additional support after discharge	medical treatment, and patients' satisfaction	*status, health-related quality of life, lifestyle, self-management, medical treatment, and patients' satisfaction to explain reduction in readmissions"*<br>- Patient assessment at discharge,<br>- Self-management program for patients<br>- Patient tailored care plan (by case manager and primary care)<br>- *IT platform* for case management to manage health records including *Web-based call center* to contact case manager.<br>- Follow up: specialized nurse and primary care team (Barcelona).

Jefford (2008) [32], RCT (1:1)	GPs taking care of cancer patients (n=97)	Baseline and 7 days (range 6-15)	Usual information without extra tax	GPs' confidence, knowledge, satisfaction, and perception	*"To examine the effectiveness of information regarding chemotherapy, potential adverse effects and recommended managements in improving GPs knowledge, confidence, satisfaction regarding communication, and shared care and perception of information received".* *Fax* was used to provide GPs with extra information about patient-, chemotherapy specific and contact information.
Lalonde (2008) [23], cluster RCT	Pharmacies (n=42)[d], pharmacists (n=101)	Baseline and 6 months	Usual care without ProFiL program	Feasibility and impact: primary outcomes: number of pharmaceutical opinions or refusals, secondary: pharmacists'	*"Assess the feasibility and impact of implementing ProFiL (to improve community pharmacists' management of*

				knowledge and satisfaction	*medication related problems), on the incidence of pharmaceutical opinions and refusals."*<br>- Community pharmacists received training, access to hospital consultation service and communica-tion network.<br>- *Fax* was used to inform community pharmacists about patients' medication and clinical information.<br>- Pharmacists could sent recommenda-tions to the specialist (standard from)
Santschi (2011) [24], cluster RCT	Pharmacies (n=42)[d], pharmacists (n=101), and chronic kidney disease	Baseline and 6 months	Usual care without ProFiL program	Change in BP, number of patients with BP controlled, number of hypertension drug related	*"To assess the impact of ProFiL (to improve community pharmacists' management of medication*

	patients (n=90)			problems, and community-pharmacist intervention	*related problems) on BP control and management of hypertension management."*<br>- Community pharmacists received training, access to hospital consultation service and communica-tion network.<br>- *Fax* was used to send community pharmacists, at baseline, a summary with clinical information (health problems, BP levels, laboratory results, medications).
Wulff (2013) [33], RCT (1:1)	Patients with colorectal cancer or highly probably diagnoses (n=280) from a hospital surgical department	Baseline and follow-up 270 days (divided in 90 day periods).	Usual care. GPs received electronic note about diagnosis and electronic discharge summary after treatment	GP evaluations and patients' contacts with GPs	*"To analyze effects of hospital-based case management on GPs' evaluation of intersectoral collaboration and information from the*

					hospital, patients contact with GPs during daytime and out of hour”<br>- Case manager informs GP about patients’ condition<br>- GPs received extra *Electronic summary message* (on top of usual information received from surgeons) regarding patients’ consult with case manager and regarding change in care when surgical department was involved.

[a]CVD: cardiovascular disease.

[b]BP: blood pressure.

[c]GP: general practitioner.

[d]RCT: randomized controlled trial.

[e]randomized group.

[f]DEM: diabetes electronic management system.

[g]EHR: electronic health record.

[h]Patients were randomized between shared and outpatient care. The nurse practitioner clinic care group was added as an additional comparative group.

[i]COPD: chronic obstructive pulmonary disease.

[j]ICT: information and communication technology.

[k]IT: information technology.
