# Supplementary material for: The Effectiveness of Information Technology-Supported Shared Care for Patients With Chronic Disease: A Systematic Review
Source: J Med Internet Res. 2017 Jun 22;19(6):e221. doi: 10.2196/jmir.7405 (PMC5500776; doi:10.2196/jmir.7405)
Supplement: Multimedia Appendix 3 [file jmir_v19i6e221_app3.pdf]

Study	Outcome measures (provider or professional, process, health or clinical and financial) including effects (significant effects are in italics)
Ciccone (2010) [26]	Health or clinical:<br>*Physical activity (days p/w): + (P<.001)*[a]<br>*Time spent on PA*[b]*: + (P<.001)*[a]<br>*+ Self efficacy, coping, to be able to access social support* [c]<br>*+ Self-monitoring: increase with extra 20-27%*[c]<br>*+ Diastolic and systolic BP*[d]*: (P<.001)*[a]<br>*+ Statistical significant change (10-20%: BMI*[e]*, LDL*[f]*, systolic BP, and total cholesterol)*[c]<br>– HDL[g,c]<br>*+ High-density lipoprotein level*[c]<br>*+ Adoption of healthy diet increase from 39.4% to 80.7%*[c]<br>*+ Vitality; average in follow-up 5.28 higher than starting score* [c]<br>*+ Satisfaction*[c]<br>Provider or professional:<br>High satisfaction from physicians and care managers relate to effective collaborate between care managers and physician[c]
Smith (2008) [31]	Process:<br>Process of diabetes care: – (*P*=.41)[h]<br>Financial:<br>*Total cost: + (P=.02)*[h]<br>*Outpatient cost: + (P=.04)*[h]<br>Health or clinical:<br>Metabolic and cardiovascular risk control<br>*Smoking cessation: + (P=.04)*[h]<br>*Aspirin use: + (P=.001)*[h]<br>– Other metabolic and cardiovascular outcomes (eg, LDL-C levels, BP, and cardio metabolic drugs)[a]
Carallo (2015) [25]	Health or clinical:<br>*HbA1c*[i]*: + (P=.01)*[a]<br>*BMI: + (P=.03)*[a]<br>*LDL cholesterol: + (P=.003)*[a]*; + (P=.001)*[j]<br>– BP, triglycerides, and waist[c]
Gurwitz (2014)	Health or clinical:<br>Number of PCP[k] visits after discharge within:

[30]	7 days: – HR: 0.95 (95% CI 0.83-1.1)[h]<br>14 days: – HR: 0.98 (95% CI 0.89-1.1)[h]<br>30 days: – HR: 0.99 (95% CI 0.91-1.1)[h]<br>Rehospitalization in 30-day period after discharge:<br>– HR for 0.94 (95% CI 0.81-1.1)[h]
DICE [27]	*Costs:*<br>– Costs mean costs per visit £8 (95% CI £5.23-£ 12.12) in usual care and £1.70 (£1.16-£2.47) in intervention[c,h]<br>Health or clinical: (metabolic control, psychosocial status, knowledge, beliefs and control, and satisfaction with treatment)<br>– Metabolic control (HbA1c, BMI, creatine, systolic BP, and diastolic BP)[c,h]<br>*+ Support for non-insulin dependent patients*[c,h]<br>– Eating problems anxiety, depression, and support (for both insulin and non-insulin dependent)[d]<br>– Support (for insulin dependent patients)[c]<br>– Knowledge[c,h]<br>*+ Belief in medical control for non-insulin dependent patients (0.5 to 6.3)*[h]<br>– Personal control, situation control, satisfaction with treatment, wellbeing for insulin and non-insulin dependent patients, and medical control for insulin dependent[c,h]<br>Satisfaction with treatment: Non-insulin dependent 95% CI −3.3 to 0.9)[c,h]; insulin dependent 95% CI −6.0 to 1.8)[c,h]<br>– Disruption of normal activities[c,h]<br>– Number of consultations and admissions[c,h]
Drummond [29]	Financial:<br>+ Integrated care saved patients 39.52 pounds sterling per year, the hospital 3.06 pounds sterling (average) per patient per year and GPs 2.41 pounds per patient per year.<br>Health or clinical:<br>Sleep disturbance:<br>– No. of nights disturbed/week: 1.01 (95% CI 0.85-1.21)[c,h]<br>– No. of days of restricted activity/month: 1.20 (95% CI 0.78-1.84)[c,h]<br>Use of bronchodilators and inhaled and oral steroids:<br>– No. of bronchodilators prescribed: 0.95 (95% CI 0.83-1.09)[c,h]<br>– No. of inhaled steroids prescribed: 0.98 (95% CI 0.88-1.09)[c,h]<br>– No of courses of oral steroids used: 0.97 (95% CI 0.79-1.20)[c,h]<br>Psychosocial outcomes:<br>Anxiety: 0 (95% CI −0.56 to 0.63)[c,h]; self-efficacy: 0 (95% CI −0.05 to 0.09)[c,h];

	living with asthma scale: 0 (95% CI −0.10 to 0.11)[c,h]; depression: 1 (95% CI 0.89-1.11)[c,h]<br>Patients' perceptions (%):<br>*+ choosing IC (P<.05)[h]; + perceiving disadvantages of IC (P<.05)[h];*<br>*+ perceiving advantages of IC (P<.05)[h];*<br>*+ perceiving attributes of GP and advantage of IC (P<.05)[h];*<br>*+ no. (%) "very satisfied" with medical care over past year (P<.05)[h]*<br>– Number of general practice consultations and hospital admissions 1.11 (95% CI 0.95 - 1.31)[c,h]<br>– No of general practice asthma consultations: 1.11 (95% CI 0.95-1.31)[c,h]<br>– No of hospital admissions for asthma: 1.31 (95% CI 0.87-1.96)[c,h]<br>*+ Hospital admissions (owning peak flow meter at start) (P<.05)[h]*<br>*+ No. of disturbed nights (owning peak flow meter at start) (P<.05)[h]*
McGhee (1994) [28]	Provider:<br>61.2% of GP[l] preferred shared care to continue and 13.6% not, 25.2% was not sure.<br>32% preferred shared care (over usual, outpatient –or NP care)<br>Process<br>+ Complete review Shared versus Outpatient care P<.001<br>+ Complete review Shared versus NP[m] clinic care P<.05<br>Financial:<br>Costs per complete review (total including patient and NHS): shared care: £80.86; Outpatient care £71.32; NP clinic care £43.67<br>Health or clinical<br>– Clinical outcomes, for example, blood pressure[c,n]<br>Shared care patients: 48.2% preference for shared care to outpatient care, 22% no preference, and 29.8% for outpatient care.
Casas<br>(2006)<br>[21]	Health or clinical:<br>*Number of readmissions: + (P=.03)[h]*<br>*Rate of readmissions: + (P=.03)[h]*<br>*Difference per year: + (P=.003)[h]*<br>*Survival without readmissions: + (P=.03)[h]*<br>Deaths (total): – (*P*=.67)[h]<br>Doctor visits (Barcelona) – (*P*=.44)[h]<br>Doctor visits (Leuven) – (*P*=.45)[h]
Garcia-Aymerich<br>(2007)	Health or clinical:<br>Clinical:

[22]	*Dyspnea + (P=.30)[h]; BMI + (P=.01)[h];* FEV_1 – (*P*=.57)[h]; FEV_1/FCV: – (*P*=.86)[h]; *Pa*O_2 (mmHg): – (*P*=.36)[h]; P*a*CO_2 (mmHg): – (*P*=.59)[h]<br>Quality of life<br>Health related quality of life: – (*P*=.56)[h]<br>Generic health-related quality of life: – (*P*=.27)[h]<br>Lifestyle:<br>Current smoking: – (*P*=.35)[h]<br>Physical activity: – (*P*=.78)[h]<br>Self-management:<br>Knowledge about:<br>*Name of disease + (P=.005)[h]; exacerbation identification: + (P<.001)[h];*<br>*early treatment of exacerbation; + (P=.04)[h]*<br>Adherence to oral treatment (MAS scale): (– *P*=.57)[h]<br>*Adherence to inhaled treatment (IAS scale): (+ P=.009)[h]*<br>Medical treatment – (*P*>.05)[h]<br>Satisfaction – (*P*=.18)[h]
Jefford<br>(2008)<br>[32]	Provider or professional:<br>*Confidence: + (P=.03)[h]*<br>Knowledge of adverse effects – (*P*=.37)[h]<br>Knowledge of reasons for referral: – (*P*=.32)[h]<br>*Satisfaction + (P=.002)[h]*<br>*Perception of information:*<br>*Usefulness of correspondence + (P<.001)[h]*<br>*Instructiveness of information + (P<.001)[h]*<br>*Easy understanding + (P<.005)[h]*<br>*Right length + (P<.001)[h]*
Lalonde<br>(2008)<br>[23]	Process:<br>– Pharmaceutical opinions prior study[c]<br>*+ Pharmaceutical opinions during the study[c]*<br>– Refusals prior and during study[c]<br>Provider or professional:<br>*+ Knowledge: 95% CI −13% to −0.5%[c,h]*<br>Satisfaction rated as “excellent”:<br>Workshop (77%)[a,c]<br>Communication-network program (23%)[a,c]<br>Consultation service (27%)[a,c]
Santschi	Health or clinical:

(2011)<br>[24]	*Systolic BP: + (P=.02)*[h]<br>Diastolic BP: – (*P*=.35)[h]<br>BP control (<130/80 mmHg): – (*P*=.13)[h]<br>Number of hypertension drug related problems at baseline (*P*=.62)[h]<br>Process:<br>*Number of written recommendations: + (P=.007)*[h]<br>*Hypertension related recommendations*<br>*+ (P=.009)*[h]
Wulff (2013)<br>[33]	Process:<br>*Number of GPs contacting hospital: +(P=.008)*[h]<br>Patient contact with GPs proportion ratio≥1 contact during daytime<br>in follow-up: – (*P*=.25)[h]<br>*Patient contact with GPs proportion ratio≥1 contact during out-of-hours:*<br>*+ (P=.02)*[h]<br>Provider or professional:<br>GPs' evaluation of received patient specific information positive effect (3 out of 20):<br>Information about psychological effects:<br>+ (*P*=.002)[h];<br>*Social effects of cancer illness: + (P=.04)*[h]<br>*GPs missed to be informed about information already given to patient by*<br>*specialist: + (P=.04)*[h]

[a]intervention group.

[b]PA: physical activity.

[c]Researchers did not provide (specific) *P*-values.

[d]BP: blood pressure.

[e]BMI: body mass index.

[f]LDL: low-density lipoprotein.

[g]HDL: high-density lipoprotein.

[h]intervention versus control group.

[i]HbA1: glycated hemoglobin.

[j]control group.

[k]PCP: primary care physician.

[l]GP: general practitioner.

[m]NP: nurse practitioner.

[n]shared care, outpatient care, and nurse practitioner clinic care.
