# Supplementary figures and images for: The Effectiveness of Information Technology-Supported Shared Care for Patients With Chronic Disease: A Systematic Review
Source: J Med Internet Res. 2017 Jun 22;19(6):e221. doi: 10.2196/jmir.7405 (PMC5500776; doi:10.2196/jmir.7405)

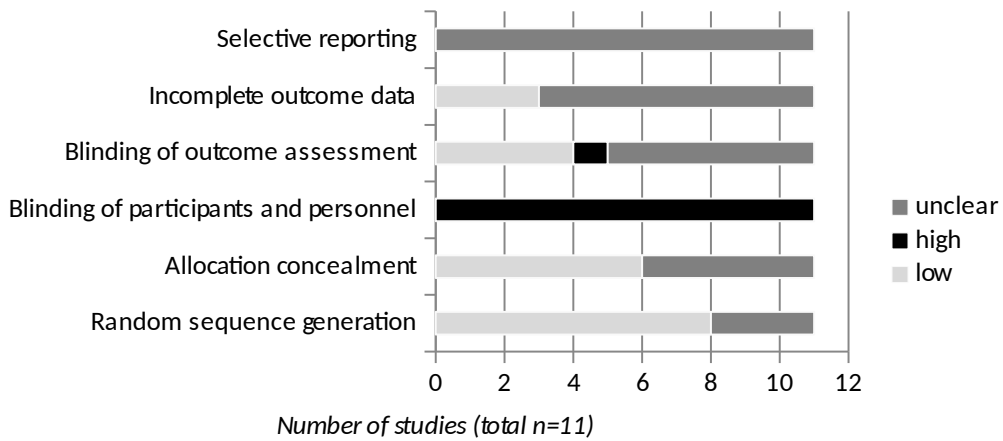

Supplement: Multimedia Appendix 4 [file jmir_v19i6e221_app4.pdf]
